# Effects of dietary oxidative balance score on diabetic nephropathy and renal function: insights from retrospective and cross-sectional studies

**DOI:** 10.3389/fnut.2025.1560913

**Published:** 2025-03-20

**Authors:** Yong Huang, Linfeng Wang, Gaojie Zhang, Yueqiang Peng, Qiao Xu, Ziling Wei, Jiang Yu, Huayang Zhang, Yao Zhang, Jiayu Liu

**Affiliations:** ^1^Department of Urology, The First Affiliated Hospital of Chongqing Medical University, Chongqing, China; ^2^Department of Urology, Chinese Academy of Medical Sciences and Peking Union Medical College, Beijing, China; ^3^Department of Urology, The Affiliated Yongchuan Hospital of Chongqing Medical University, Chongqing, China; ^4^School of Psychiatry, The First Clinical College of Chongqing Medical University, Chongqing, China; ^5^Department of Urology, Chongqing Western Hospital, Chongqing, China

**Keywords:** dietary oxidative balance score, diabetic nephropathy, renal function, chronic inflammation, NHANES

## Abstract

**Background:**

The relationship between dietary oxidative balance score (DOBS) and diabetes-related renal events remains unclear.

**Methods:**

In this study, the associations between serum micronutrients and diabetic nephropathy (DN) in participants matched by propensity score (PSM) were retrospectively analyzed. And next, a cross-sectional analysis was performed with the National Health and Nutritional Examination Survey (NHANES) database. Weighted multivariate adjusted logistic regression models, dose–response curves, subgroup analysis, and mediation analysis were the main methods of this study. Finally, sensitivity analyses were performed by PSM and multiple imputation (MI).

**Results:**

Retrospective findings suggest that single antioxidants may not be representative of an individual’s overall antioxidant levels. The results of the cross-sectional study indicated that the higher the DOBS, the greater the beneficial effects on DN [Q4 vs. Q1: OR (95% CI): 0.78 (0.63, 0.96), *p* for trend = 0.008] and renal function in DN [Q4 vs. Q1: *β* (95% CI): 5.395 (1.590, 9.199), *p* for trend = 0.004]. The above correlations were linear negative correlation (*p* for nonlinear = 0.989) and linear positive correlation (*p* for nonlinear = 0.593) respectively. Chronic inflammation mediated the above associations to some extent. The results of sensitivity analysis were consistent with the original analysis.

**Conclusion:**

Higher dietary antioxidant exposure has a positive effect on DN and renal function in DN, mediated partially by chronic inflammation.

## 1 Introduction

The global health burden of diabetes on individuals, families, and nations continues to escalate. According to the 2021 International Diabetes Federation (IDF) Diabetes Atlas, approximately 537 million (10.5%) adults aged 20–79 worldwide currently live with diabetes, a figure projected to rise to 783 million by 2045 (1). Among its most severe complications, diabetic nephropathy (DN) is defined as the coexistence of diabetes and chronic kidney disease (CKD) in the absence of other identifiable causes of renal injury. CKD itself is characterized by kidney damage persisting for ≥3 months, evidenced by reduced glomerular filtration rate (GFR < 60 mL/min/1.73m^2^) or increased albuminuria (2). Epidemiological studies indicate that 20–40% of individuals with diabetes develop DN (3), which now constitutes a leading etiology of end-stage renal disease (ESRD), affecting approximately 40% of type 2 and 30% of type 1 diabetes patients (4, 5).

Oxidative stress (OS), a pivotal pathophysiological mechanism in DN, arises from dynamic imbalances between pro-oxidant factors and antioxidant defense systems (6). While preclinical evidence demonstrates that antioxidants mitigate diabetes and its complications by suppressing OS and reducing free radical production (7), their clinical utility in DN management remains controversial. Existing clinical trials report conflicting conclusions regarding the association between antioxidant interventions and DN progression (8–10), potentially attributable to the predominant focus on isolated antioxidant agents. This observation prompted our hypothesis: the biological effects of single antioxidants may inadequately reflect an individual’s holistic antioxidant capacity.

To address this research gap, we introduced an innovative analytical framework. The Dietary Oxidative Balance Score (DOBS), a novel dietary assessment tool, systematically quantifies the dynamic equilibrium between pro-oxidant and antioxidant components in diets, with established associations to biological aging and periodontal health (11, 12). Although prior studies have explored DN correlations using the Composite Dietary Antioxidant Index (CDAI) (13), DOBS encompassing a broader spectrum of dietary constituents remains uninvestigated in the context of DN and its renal function outcomes.

This study employs a three-phase analytical framework: First, a retrospective analysis preliminarily explores associations between serum trace elements and DN-related renal dysfunction. Second, cross-sectional analyses elucidate dose–response relationships between DOBS and the prevalence of DN and its renal function. Finally, mediation analyses probe the potential role of chronic inflammation in these associations.

## 2 Materials and methods

### 2.1 Overall study design and data source

This study employed a two-phase analytical approach. Initially, a retrospective analysis was conducted utilizing data from the Physical Examination Center of our institution, spanning January 2023 to December 2023. Subsequently, a cross-sectional analysis was performed using data from the National Health and Nutritional Examination Survey (NHANES) database.

The retrospective component of this investigation received ethical approval from the Institutional Review Board of the First Affiliated Hospital of Chongqing Medical University on June 11, 2024 (Ethics Approval Number: 2024–032-01; ChiCTR2400086297). For the NHANES component, as the database had already obtained approval from the National Center for Health Statistics (NCHS) Research Ethics Review Board, no additional ethical clearance was required from our institution. All participants had signed an informed consent form.

### 2.2 Retrospective analysis based on hospital physical examination data

**Figure 1:**
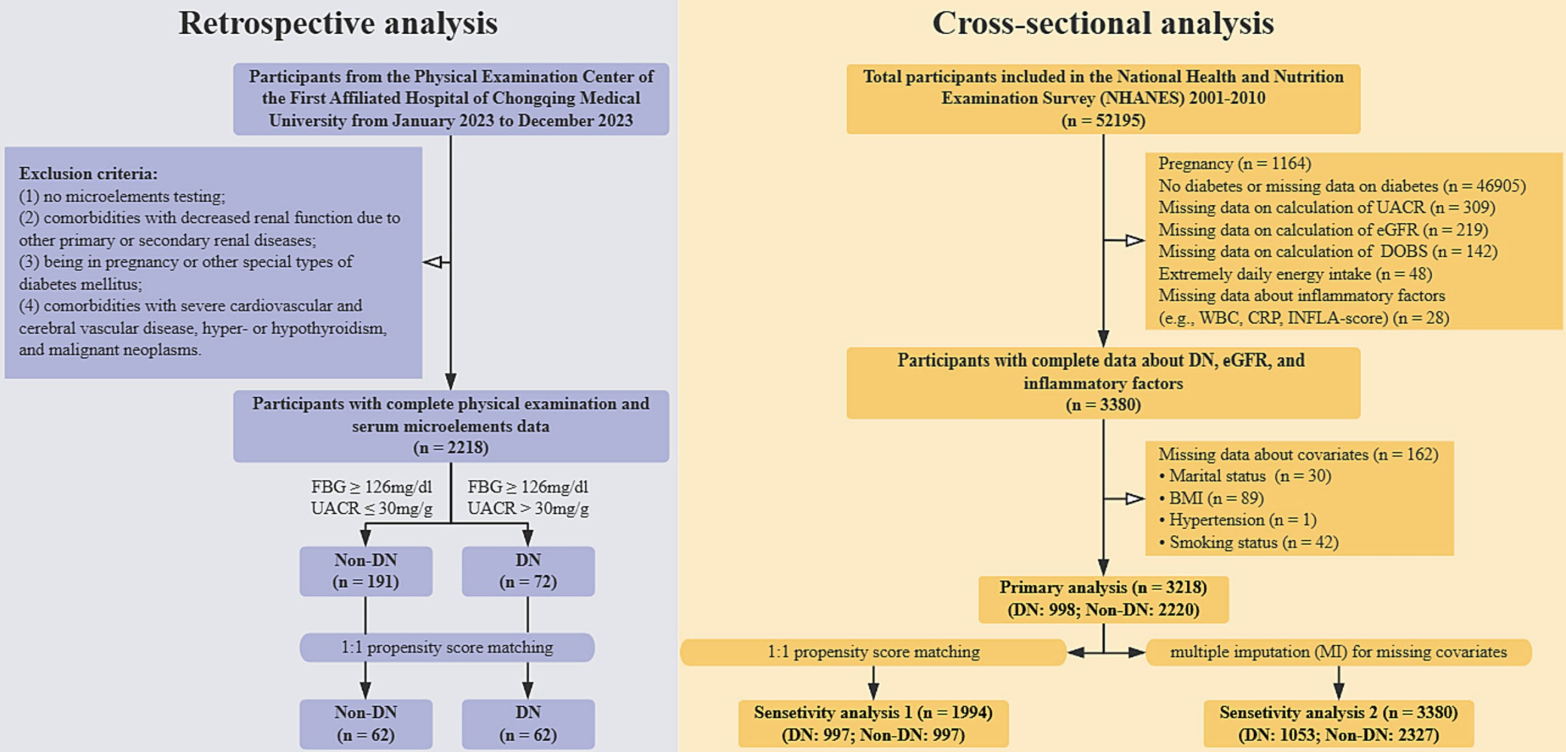
The general flowchart of this study. DOBS, Dietary oxidative balance score; DN, Diabetic nephropathy; UACR, Urine albumin-to-creatinine ratio; eGFR, estimated glomerular filtration rate; WBC, White blood cell; CRP, C-reactive protein; INFLA-score, Low-grade inflammation score; BMI, Body mass index.

The exclusion criteria were defined as follows: (1) absence of microelement testing; (2) comorbidities involving impaired renal function due to primary or secondary renal diseases; (3) pregnancy or presence of other specific types of diabetes mellitus; and (4) comorbidities with severe cardiovascular or cerebrovascular diseases, hyper- or hypothyroidism, or malignant neoplasms. Participants were stratified into a DN group and a non-DN group based on predefined thresholds for FBG ≥ 126 mg/dL and UACR ≥30 mg/g. To ensure comparability, baseline characteristics between the two groups were balanced using 1:1 propensity score matching (PSM). Following this process, a total of 124 participants were enrolled in each group (Figure 1).

### 2.3 Cross-sectional analysis in NHANES

#### 2.3.1 Study population

A total of 5 cycles of data from NHANES 2001–2010 were incorporated. The specific strategies used to exclude participants were as follows: (1) Pregnancy status (*n* = 1,164); (2) Non-diabetes or missing data on diagnosis of diabetes (*n* = 46,905); (3) Missing data on the calculation of UACR (*n* = 309); (4) Missing data on calculation of estimated glomerular filtration rate (eGFR) (*n* = 219); (5) Missing data on intake of dietary antioxidants or pro-oxidants (*n* = 142); (6) Extremely energy intake (< 500 kcal/day or > 5,000 kcal/day) (*n* = 48); (14) (7) Missing data on inflammatory factors (*n* = 28); (8) Missing data on covariates (*n* = 162). Ultimately, a total of 3,218 participants were enrolled in this study (Figure 1).

Data from 5 cycles of the NHANES spanning 2001 to 2010 were included in this study. The following exclusion criteria were applied sequentially: (1) pregnancy (*n* = 1,164); (2) absence of diabetes or missing diabetes diagnosis data (*n* = 46,905); (3) missing UACR data (n = 309); (4) missing estimated glomerular filtration rate (eGFR) data (*n* = 219); (5) missing dietary antioxidant or pro-oxidant intake data (*n* = 142); (6) extreme energy intake (< 500 kcal/day or > 5,000 kcal/day) (*n* = 48); (7) missing inflammatory factor data (*n* = 28); and (8) missing covariate data (*n* = 162). After applying these exclusion criteria, a final cohort of 3,218 participants was included in the analysis (Figure 1).

#### 2.3.2 Evaluation of DOBS

Dietary intake was assessed using 24-h dietary recall records. Each participant was eligible for two interview sessions. For participants with two complete dietary recall interviews, the mean value of the two sessions was calculated to represent their dietary intake. In cases where only one interview was completed, the data from the first session were used. The DOBS was calculated based on the intake of 14 dietary antioxidants and 2 dietary pro-oxidants (12), as detailed in Supplementary Tables 1, 2. The individual scores for these 16 components were summed to derive the DOBS, with higher scores indicating greater antioxidant levels.

#### 2.3.3 Evaluation of DN and renal function

In this study, criteria for the diagnosis of diabetes were as follows: (1) FBG ≥ 126 mg/dL; (2) glycosylated hemoglobin ≥6.5%; (3) doctor told you have diabetes; (4) 2-h oral glucose tolerance test (OGTT) ≥ 200 mg/dL; (5) oral hypoglycemic drugs or use insulin. Those who met any of these criteria were considered to be diabetic. Diabetic patients with a UACR >30 mg/g were recognized as DN (15). Then, the Chronic Kidney Disease Epidemiology Collaboration (CKD-EPI) formula was utilized to calculate eGFR, which can be completed using the “CKDEpi.creat” package of the R software (16). It took into account the participant’s serum creatinine (Scr), age, gender and race (17). The details were as follows:


$$\begin{array}{c} \text{eGFR}=141\times \min{\left(Scr/\kappa ,1\right)}^{\alpha }\times \max{\left(Scr/\kappa ,1\right)}^{-1.209}\\ \times 0.993^{\text{age}}\times 1.018\;\left[\text{if}\;\text{female}\right]\times 1.159\;\left[\text{if}\;\text{black}\right]\text{.} \end{array}$$

#### 2.3.4 Evaluation of covariates

Based on previous studies (13, 15), variance inflation factors (VIF) < 10 were considered to be free of multicollinearity (18), the following covariates (age, gender, race, marital status, BMI, hypertension and smoking status) were finally included in this study. Hypertension was recognized as having a systolic blood pressure (SBP) ≥ 130 mmHg or diastolic blood pressure (DBP) ≥ 80 mmHg or being on antihypertensive medications. Smoking status was categorized as non-smoker, previous smoker or current smoker.

#### 2.3.5 Evaluation of inflammatory mediators

To investigate the potential mediating role of inflammation in the relationship between exposure and outcome, this study incorporated several laboratory indicators as markers of inflammation. These included white blood cell count (WBC), lymphocyte count (L), monocyte count (M), neutrophil count (N), and C-reactive protein (CRP). Additionally, a composite indicator, the low-grade inflammation score (INFLA-score), was utilized. The specific calculation methodology for the INFLA-score is detailed in Supplementary Table 1. Briefly, the scores for each of the four parameters were summed to derive the INFLA-score, which ranges from −16 to 16. Higher INFLA-scores indicate elevated levels of low-grade inflammation (19).

### 2.4 Statistical analysis

In the retrospective analysis, continuous variables were summarized as mean ± standard deviation (SD) for normally distributed data or median (interquartile range) for non-normally distributed data. Categorical variables were expressed as percentages. Statistical analyses included the independent samples *t*-test for normally distributed continuous variables, the Mann–Whitney *U* test for non-normally distributed continuous variables, and the chi-square test for categorical variables. Correlation analyses were performed using Pearson’s or Spearman’s methods, depending on data distribution. Both univariate and multivariate logistic regression analyses were conducted to assess associations between variables.

Considering the complex sampling design of NHANES and in order to make the sample nationally representative, the specific rules for weight use were as follows: (1) As mentioned before, if the participants had two complete dietary data, the dietary two-day sample weight (WTDR2D) was used as a weight, otherwise the dietary day one sample weight (WTDRD1) was used as a weight. (2) Data from NHANES 2001–2010 for a total of 5 cycles were included, so the final weight used was either WTDRD1 * 1/5 or WTDR2D * 1/5.

In the cross-sectional analysis, participants’ baseline characteristics were first described. The relationship between the DOBS and DN, as well as eGFR in DN, was examined using multivariable-adjusted regression models and stratified analyses. To explore the dose–response relationship between DOBS and outcomes, restricted cubic spline (RCS) analysis and threshold effects analysis were employed. Additionally, an exploratory mediation analysis was conducted using the “mediation” package in R software to investigate the potential mediating role of inflammation in the relationship between DOBS and outcomes. To ensure the robustness of the findings, two sensitivity analyses were performed. First, 1:1 PSM was conducted using the “MatchIt” package in R software. Second, multiple imputation (MI) was applied using the “mice” package in R software to address missing data. New datasets were generated through these methods, and the aforementioned analyses were repeated to verify result stability (Figure 1).

All statistical analyses in this study were performed with the help of R software (version 4.2.1), SPSS 26.0 and EmpowerStat software (version 4.1). The criterion for statistical significance was *p* < 0.05.

## 3 Results

### 3.1 Retrospective analysis

Supplementary Table 3 demonstrated the clinical baseline data for the two groups of participants after PSM. Regarding serum microelements, only serum selenium was considered to be an independent protective factor for DN [Odds ratio (OR) (95% CI), *p*: 0.764 (0.638, 0.917), 0.004] (Supplementary Table 4). In addition, Supplementary Table 5 exhibited the correlation between serum microelements and renal function in DN, and it could be found that serum iron, zinc, and selenium were positively correlated with renal function in DN (All *r* > 0 and *p* < 0.05).

### 3.2 Cross-sectional analysis in NHANES

#### 3.2.1 Weighted baseline characteristics of participants

Table 1 demonstrates the weighted baseline characteristics of the participants after DOBS categorization through quartiles. It could be found that the higher the DOBS, the lower the prevalence of DN and the higher the eGFR in DN (*p* < 0.001 and *p* = 0.008, respectively). Additionally, it was interesting to note that this study also observed a greater reduction in WBC, N, CRP, and INFLA-score in the higher DOBS group (All *p* < 0.001). A total of 1994 participants (997 with DN and 997 without DN) were included after PSM. A schematic diagram of the balance of the dataset before and after PSM was shown in Supplementary Figure 1. Supplementary Table 6 shows the specific details of the weighted baseline characteristics of the participants after PSM, which were consistent with the primary analysis.

**Table 1:** Weighted baseline characteristics of participants before PSM.

Characteristics	All	DOBS				*p*
		Q1	Q2	Q3	Q4	
Continuous variables (Mean ± SD)						
DOBS	16.81 ± 6.61	7.32 ± 1.95	13.12 ± 1.42	18.56 ± 1.69	24.66 ± 1.98	< 0.001
Age, years	58.96 ± 13.80	59.98 ± 14.20	59.42 ± 13.77	58.83 ± 13.76	58.01 ± 13.49	0.029
BMI, kg/m^2^	32.67 ± 7.30	32.27 ± 6.85	32.29 ± 7.34	33.48 ± 7.67	32.46 ± 7.14	0.001
Ualb, ug/ml	129.35 ± 808.94	166.05 ± 838.28	150.39 ± 1190.31	134.52 ± 697.63	82.21 ± 460.95	0.167
Ucr, mg/dl	117.27 ± 71.15	119.22 ± 78.18	115.70 ± 74.45	120.87 ± 72.74	113.49 ± 60.76	0.120
UACR, mg/g	118.20 ± 640.46	169.94 ± 738.51	135.30 ± 858.93	103.43 ± 543.30	82.71 ± 421.91	0.041
Scr, mg/dl	0.97 ± 0.40	1.02 ± 0.50	0.99 ± 0.51	0.95 ± 0.31	0.93 ± 0.27	< 0.001
eGFR, mL/min/1.73m^2^	82.50 ± 23.99	80.43 ± 25.81	82.56 ± 25.49	81.95 ± 23.37	84.47 ± 21.84	0.008
Dietary fiber, g/day	16.01 ± 8.43	8.91 ± 3.42	12.75 ± 4.51	16.20 ± 6.01	23.37 ± 9.34	< 0.001
Carotene, ug/day	2523.76 ± 3493.76	1361.05 ± 2344.01	2123.88 ± 2927.88	2387.14 ± 2829.70	3792.27 ± 4597.95	< 0.001
Vitamin B2, mg/day	2.08 ± 0.97	1.72 ± 0.59	2.13 ± 0.71	2.91 ± 1.02	1.72 ± 0.59	< 0.001
Niacin, mg/day	22.95 ± 10.40	19.08 ± 6.68	23.70 ± 7.73	31.55 ± 10.98	19.08 ± 6.68	< 0.001
Vitamin B6, mg/day	1.87 ± 0.98	1.48 ± 0.51	1.91 ± 0.64	2.74 ± 1.11	1.48 ± 0.51	< 0.001
Total folate intake, ug/day	381.18 ± 184.80	215.27 ± 72.58	308.84 ± 105.18	387.65 ± 122.11	548.13 ± 198.44	< 0.001
Vitamin B12, ug/day	5.34 ± 6.59	2.65 ± 1.74	4.16 ± 4.14	5.55 ± 7.69	7.96 ± 7.98	< 0.001
Vitamin C, mg/day	79.72 ± 70.93	44.59 ± 44.58	65.97 ± 56.69	78.56 ± 63.26	116.37 ± 84.91	< 0.001
Vitamin E, mg/day	6.82 ± 4.56	3.61 ± 1.67	5.27 ± 2.21	6.93 ± 3.34	10.19 ± 5.88	< 0.001
Calcium, mg/day	835.84 ± 440.30	477.42 ± 210.40	664.27 ± 289.33	861.57 ± 328.53	1196.34 ± 472.07	< 0.001
Magnesium, mg/day	273.54 ± 114.84	156.70 ± 41.38	220.99 ± 48.35	283.97 ± 72.96	386.55 ± 111.71	< 0.001
Zinc, mg/day	11.66 ± 8.17	6.63 ± 2.59	9.31 ± 4.17	11.59 ± 4.70	17.11 ± 11.78	< 0.001
Copper, mg/day	1.29 ± 1.05	0.74 ± 0.24	1.04 ± 0.60	1.37 ± 1.29	1.78 ± 1.17	< 0.001
Selenium, ug/day	104.96 ± 48.25	66.53 ± 22.68	87.58 ± 29.32	108.84 ± 37.92	141.77 ± 54.24	< 0.001
Total fat intake, g/day	75.03 ± 37.24	49.69 ± 21.94	64.08 ± 25.64	78.36 ± 30.89	98.16 ± 43.69	< 0.001
Iron, mg/day	15.03 v 7.38	8.92 ± 3.23	12.15 ± 4.33	15.12 ± 4.83	21.47 ± 8.30	< 0.001
Total energy intake, kcal/day	1879.94 ± 730.43	1281.82 ± 430.38	1610.10 ± 469.21	1946.75 ± 558.52	2445.85 ± 773.69	< 0.001
WBC, 1000 cells/ul	7.73 ± 2.47	7.90 ± 2.37	7.74 ± 3.01	7.91 ± 2.44	7.41 ± 2.04	< 0.001
Lymphocyte, 1000 cells/ul	2.25 ± 1.34	2.26 ± 0.86	2.28 ± 2.09	2.32 ± 1.31	2.15 ± 0.83	0.052
Monocyte, 1000 cells/ul	0.58 ± 0.20	0.57 ± 0.19	0.58 ± 0.21	0.59 ± 0.21	0.57 ± 0.19	0.248
Neutrophils, 1000 cells/ul	4.63 ± 1.73	4.79 ± 1.88	4.63 ± 1.80	4.72 ± 1.68	4.42 ± 1.57	< 0.001
Eosinophils, 1000 cells/ul	0.22 ± 0.16	0.23 ± 0.18	0.21 ± 0.16	0.23 ± 0.16	0.23 ± 0.16	0.005
Basophils, 1000 cells/ul	0.04 ± 0.06	0.05 ± 0.05	0.04 ± 0.06	0.05 ± 0.06	0.04 ± 0.06	< 0.001
G/L	2.47 ± 1.32	2.52 ± 1.34	2.50 ± 1.49	2.46 ± 1.19	2.44 ± 1.28	0.655
Platelet	260.40 ± 76.44	263.28 ± 77.76	261.13 ± 84.26	262.58 ± 76.92	255.65 ± 68.24	0.147
CRP, mg/dl	0.65 ± 1.07	0.79 ± 1.56	0.69 ± 1.10	0.60 ± 0.82	0.56 ± 0.79	< 0.001
INFLA-score	0.75 ± 6.21	1.32 ± 6.13	0.64 ± 6.07	1.05 ± 6.18	0.12 ± 6.33	< 0.001
Categorical variables (%)						
Gender						0.202
Male	51.41	50.15	51.53	49.44	54.13	
Female	48.59	49.85	48.47	50.56	45.87	
Race						< 0.001
Mexican American	9.53	10.31	8.97	9.52	9.39	
Non-Hispanic Black	15.56	24.81	18.20	12.22	10.20	
Non Hispanic White	64.07	53.49	60.15	68.82	69.96	
Others	10.84	11.39	12.67	9.44	10.45	
Marital status						0.086
Never married	8.74	9.32	9.38	8.64	7.95	
Married/living with partner	62.45	60.74	60.32	60.90	66.80	
Windowed/divorced/separated	28.80	29.94	30.30	30.46	25.25	
Hypertension						0.001
No	17.04	15.18	16.04	14.95	21.17	
Yes	82.96	84.82	83.96	85.05	78.83	
Smoking status						< 0.001
Non-smokers	45.63	40.11	40.69	48.66	50.30	
Former smoker	35.22	33.14	37.08	35.39	35.17	
Current smoker	19.15	26.74	22.23	15.96	14.52	
Diabetic nephropathy						< 0.001
No	72.56	66.81	70.84	75.77	74.83	
Yes	27.44	33.19	29.16	24.23	25.17	

DOBS, Dietary oxidative balance score; Ualb, Urine albumin; Ucr, Urine creatinine; UACR, Urine albumin-to-creatinine ratio; Scr, Serum creatinine; eGFR, estimated glomerular filtration rate; INFLA-score, Low-grade inflammation score; PSM, Propensity score matching.

#### 3.2.2 Relationship between DOBS and DN and its renal function

The relationships between DOBS and DN and eGFR in DN were demonstrated in Tables 2, 3, respectively. Under the fully adjusted model (Model 3), increased DOBS was associated with reduced prevalence of DN [OR (95% CI), *p*: 0.98 (0.97, 1.00), 0.006; *p* for trend = 0.008] and improved eGFR in DN [*β* (95% CI), *p*: 0.254 (0.047, 0.461), 0.016; *p* for trend = 0.004]. Further, to assess whether missing covariates affected the research results, 5 new datasets were obtained in this study through MI. The above analysis was repeated and the effect values were combined. The detailed results were exhibited in Supplementary Table 7. Moreover, the correlations between DOBS and DN and eGFR in DN under different grouping conditions before and after PSM were shown in Supplementary Tables 8, 9, respectively. It could be seen that the association between DOBS and the two outcomes remained stable across subgroups (All *p* for interaction >0.05).

**Table 2:** The relationship between DOBS and DN.

Exposure	OR (95% CI), *p*		
	Model 1	Model 2	Model 3
Before PSM			
DOBS	0.98 (0.97, 0.99), < 0.001	0.98 (0.97, 0.99), 0.003	0.98 (0.97, 1.00), 0.006
DOBS (Categorical)			
Q1	Ref	Ref	Ref
Q2	0.85 (0.69, 1.05), 0.128	0.87 (0.70, 1.07), 0.186	0.87 (0.70, 1.08), 0.206
Q3	0.71 (0.58, 0.88), 0.001	0.74 (0.60, 0.92), 0.006	0.75 (0.61, 0.93), 0.008
Q4	0.71 (0.57, 0.87), 0.001	0.76 (0.62, 0.94), 0.013	0.78 (0.63, 0.96), 0.021
*p* for trend	< 0.001	0.005	0.008
After PSM			
DOBS	0.99 (0.97, 1.00), 0.034	0.99 (0.97, 1.00), 0.042	0.99 (0.97, 1.00), 0.038
DOBS (Categorical)			
Q1	Ref	Ref	Ref
Q2	0.82 (0.64, 1.06), 0.132	0.81 (0.63, 1.05), 0.115	0.82 (0.63, 1.06), 0.123
Q3	0.76 (0.60, 0.98), 0.031	0.77 (0.60, 0.99), 0.038	0.77 (0.60, 0.99), 0.041
Q4	0.78 (0.61, 0.99), 0.042	0.78 (0.60, 1.00), 0.048	0.77 (0.60, 0.99), 0.044
*p* for trend	0.035	0.044	0.041

Model 1 was a crude model; Model 2 adjusted for age, gender and race; Model 3 was a fully adjusted model, which adjusted for marital status, BMI, hypertension and smoking status based on model 2. DOBS, Dietary oxidative balance score; DN, Diabetic nephropathy; PSM, Propensity score matching; OR, Odds ratio.

**Table 3:** The relationship between DOBS and eGFR in DN.

Exposure	*β* (95% CI), *p*		
	Model 1	Model 2	Model 3
Before PSM			
DOBS	0.404 (0.149, 0.659), 0.002	0.215 (0.006, 0.423), 0.044	0.254 (0.047, 0.461), 0.016
DOBS (Categorical)			
Q1	Ref	Ref	Ref
Q2	1.434 (−3.456, 6.325), 0.566	0.220 (−3.705, 4.146), 0.912	0.700 (−3.186, 4.586), 0.724
Q3	4.159 (−0.621, 8.939), 0.088	1.158 (−2.719, 5.035), 0.558	2.026 (−1.826, 5.879), 0.303
Q4	7.842 (3.130, 12.554), 0.001	4.724 (0.887, 8.562), 0.016	5.395 (1.590, 9.199), 0.006
*p* for trend	< 0.001	0.014	0.004
After PSM			
DOBS	0.403 (0.149, 0.657), 0.002	0.203 (−0.005, 0.411), 0.056	0.238 (0.031, 0.444), 0.024
DOBS (Categorical)			
Q1	Ref	Ref	Ref
Q2	0.863 (−4.153, 5.879), 0.736	−0.293 (−4.326, 3.739), 0.887	0.025 (−3.973, 4.022), 0.990
Q3	2.874 (−1.956, 7.704), 0.244	0.082 (−3.832, 3.995), 0.967	0.971 (−2.919, 4.862), 0.625
Q4	8.134 (3.442, 12.827), < 0.001	4.423 (0.591, 8.256), 0.024	4.929 (1.124, 8.733), 0.011
*p* for trend	< 0.001	0.022	0.009

Model 1 was a crude model; Model 2 adjusted for age, gender and race; Model 3 was a fully adjusted model, which adjusted for marital status, BMI, hypertension and smoking status based on model 2. DOBS, Dietary oxidative balance score; eGFR, estimated glomerular filtration rate; DN, Diabetic nephropathy; PSM, Propensity score matching; OR, Odds ratio.

**Figure 2:**
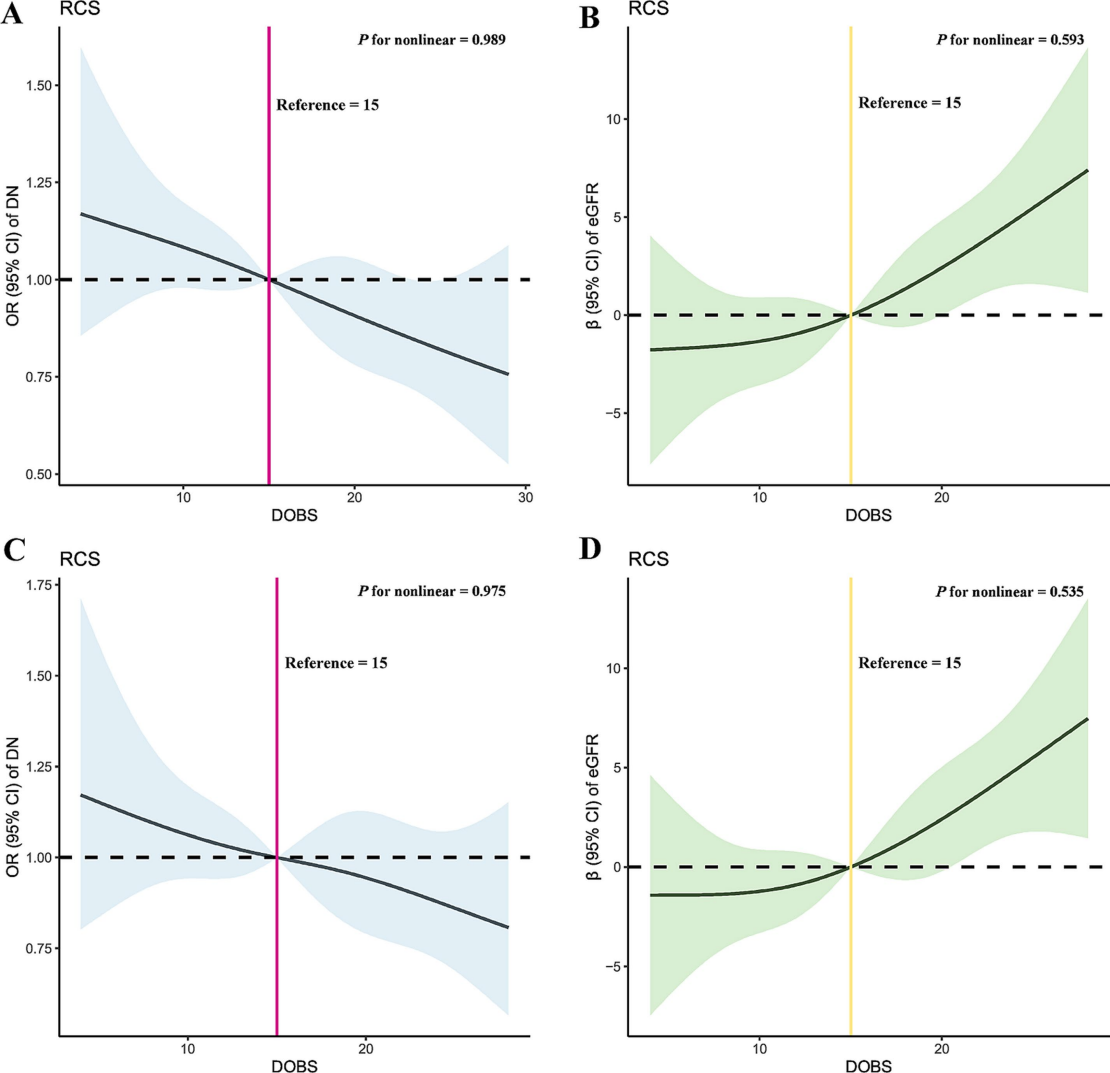
Dose–response relationship between DOBS and DN and eGFR in DN before and after PSM through RCS. **(A)** RCS of DOBS and DN before PSM; **(B)** RCS of DOBS and eGFR in DN before PSM; **(C)** RCS of DOBS and DN after PSM; **(D)** RCS of DOBS and eGFR in DN after PSM. DOBS, Dietary oxidative balance score; DN, Diabetic nephropathy; eGFR, estimated glomerular filtration rate; RCS, Restricted cubic splines; PSM, Propensity score matching; OR, Odds ratio.

Figure 2 demonstrates the results of RCS in this study. Taking the DOBS values at OR = 1 and *β* = 0 as reference values, the relationships between DOBS and DN (*p* for nonlinear = 0.989) and eGFR in DN (*p* for nonlinear = 0.593) could be considered as linear. Furthermore, the results of the threshold effects analysis similarly indicated that the effects of DOBS with DN (log likelihood ratio = 0.095) and eGFR in DN (log likelihood ratio = 0.106) both were appropriate to be explained by a single-line model (Supplementary Table 10). These results were consistent across sensitivity analyses.

#### 3.2.3 Exploratory inflammatory mediation analysis

**Figure 3:**
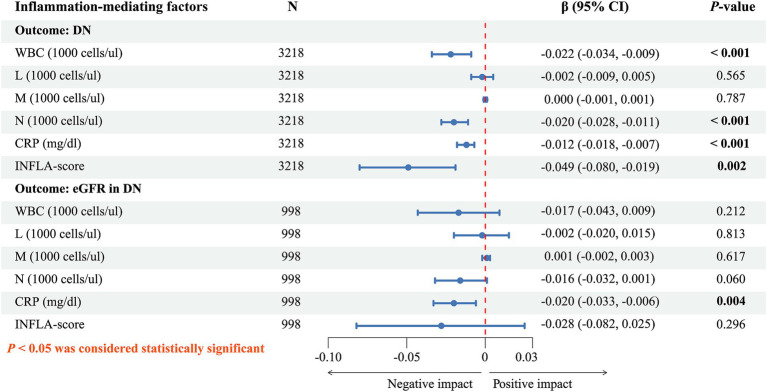
The relationship between DOBS and inflammation-mediating factors. DOBS, Dietary oxidative balance score; WBC, White blood cell; L, Lymphocyte; M, Monocyte; N, Neutrophil; CRP, C-reactive protein; INFLA-score, Low-grade inflammation score.

**Figure 4:**
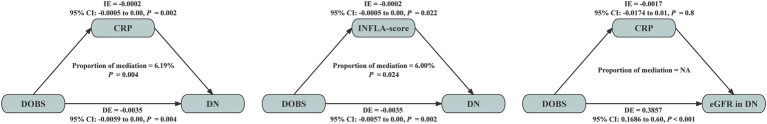
Mediation analyses with inflammatory factors between the association of DOBS and DN and eGFR in DN. DOBS, Dietary oxidative balance score; DN, Diabetic nephropathy; CRP, C-reactive protein; INFLA-score, Low-grade inflammation score; IE, Indirect effect; DE, Direct effect.

Under the fully adjusted model (model 3), WBC (*β*, *p*: −0.022, < 0.001), N (*β*, *p*: −0.020, < 0.001), CRP (*β*, *p*: −0.012, < 0.001) and INFLA-score (*β*, *p*: −0.049, 0.002) were further employed for exploratory mediation analysis between DOBS and DN (Figure 3). In addition, CRP (*β*, *p*: −0.020, 0.004) was further employed for exploratory mediation analysis between DOBS and eGFR in DN (Figure 3). The results of the exploratory mediation analysis presented in Figure 4 indicate that CRP and INFLA-score mediated the association between DOBS and DN to some extent, with the mediation proportions of 6.19 and 6.00%, respectively.

## 4 Discussion

In a preliminary retrospective analysis, this study identified a protective effect of serum selenium against DN, which contrasts with previous findings (20). Given this discrepancy, we hypothesized that a single antioxidant may not adequately represent an individual’s overall antioxidant status. Furthermore, dietary micronutrient intake is the primary source of serum micronutrients. Motivated by these considerations, this study introduced the DOBS as a comprehensive index to assess the balance between dietary oxidative and antioxidant components.

To our knowledge, this is the first study to investigate the relationship between the DOBS and DN, as well as renal function in DN. In the cross-sectional analysis, higher DOBS levels were associated with a reduced prevalence of DN and improved renal function in DN, as demonstrated by the fully adjusted model. Specifically, DOBS exhibited a linear negative association with DN prevalence and a linear positive association with renal function. Stratified analyses confirmed that these relationships remained stable across various subgroups. The consistency of these findings was further validated in new datasets generated through PSM and MI, underscoring the robustness of the results. Additionally, mediation analyses revealed that CRP and the INFLA-score partially mediated the association between DOBS and DN.

Emerging evidence indicates that the pathogenesis of DN involves convergent pathways where diverse initiating factors ultimately activate common downstream effectors. This pathophysiological cascade prominently features sustained overproduction of pro-inflammatory cytokines [e.g., interleukin 6 (IL-6), tumor nucrosis factor (TNF-*α*)] and fibrogenic mediators [e.g., transforming growth factor-*β* (TGF-*β*)]. Crucially, oxidative stress serves as both a principal instigator and amplifier in this process, driving disease progression through reactive oxygen species (ROS)-mediated activation of nuclear factor κB (NF-κB) signaling pathway (21). Previous studies have highlighted the beneficial effects of dietary antioxidants on diabetes and its complications. For instance, astaxanthin has been shown to protect pancreatic *β*-cells and various organs, including the kidneys, from oxidative damage associated with diabetes (22, 23). Additionally, a study by Noonin et al. demonstrated that curcumin mitigates high glucose-induced renal cell secretions, such as intracellular ROS and TGF-β, thereby inhibiting the stimulatory effects on renal fibroblasts (24). A review by Gerardo et al. further summarized the positive impacts of various dietary antioxidants on OS and antioxidant responsiveness in DN (25). Consistent with the above findings, the present study utilized DOBS, a comprehensive dietary antioxidant index, and found that higher DOBS implies greater antioxidant capacity, which in turn has a greater beneficial effect on DN and renal function in DN.

Additionally, this study revealed that chronic inflammation partially mediates the association between the Dietary Oxidative Balance Score (DOBS) and diabetic nephropathy (DN). Inflammation plays a critical role in various pathological processes in DN, including renal tubular fibrosis, inflammatory cell infiltration, extracellular matrix accumulation, and podocyte autophagy. Key inflammatory pathways, such as PI3K/AKT, JAK/STAT3, TLR/NF-κB, TNF-*α*, TGF-β1, can exacerbate structural damage in the kidneys of DN patients, ultimately contributing to renal insufficiency (26).

Numerous studies have investigated the effects of individual micronutrients on health outcomes. For instance, a randomized controlled trial demonstrated that selenium supplementation increased total antioxidant capacity (TAC) in patients with DN (27). Additionally, a selenium-deficient diet was shown to induce renal OS and injury through TGF-β1 in both normal and diabetic rats (28). Furthermore, higher dietary calcium intake has been associated with a reduced risk of type 2 diabetes (29). Regarding vitamin C, studies have indicated that type 2 diabetics with more severe DN exhibit lower vitamin C levels (30). Vitamin C may exert beneficial effects on DN by modulating blood glucose levels, regulating the expression of oxidative enzymes, and influencing inflammatory proteins in diabetic kidneys, thereby reducing both hyperglycemia and inflammatory responses (31). Similarly, dietary supplementation with pyridoxamine, a structural analog of vitamin B6, has been found to reduce advanced glycation end products (AGEs), thereby inhibiting the activation of the NF-κB and Rho/ROCK pathways, which are implicated in fibrosis and inflammatory responses (32). In contrast to these single-nutrient studies, this study introduces a comprehensive indicator by integrating 14 antioxidants and 2 pro-oxidants into the DOBS. This approach may more accurately reflect the body’s overall antioxidant status compared to individual micronutrient measurements. However, the specific mechanisms underlying these effects warrant further exploration and elucidation in future studies.

The present study has some advantages. First, this study drew inspiration from the retrospective analysis and then applied the index DOBS, which may be able to better reflect dietary antioxidant levels. Second, the robustness of the results of the present study was demonstrated by multiple sensitivity analysis methods. Finally, the role played by chronic inflammation between DOBS and DN was preliminarily explored.

This study similarly has some limitations. (1) The hospital physical examination center did not directly contain data on the dietary microelements of the participants, so serum microelements were used. And the sample size needs to be further expanded. (2) The diagnosis for DN was based on diabetes and UACR, and there was not enough time for its observation (> 3 months). (3) Due to the limited clinical information contained in NHANES, the present study was unable to further categorize DN according to the type of diabetes. (4) Some of the variables included in this study were derived from the questionnaire provided by NHANES, which introduces some recall bias. (5) The mean BMI of the patients included in this study was 32.6, which is in the class I obesity range. Considering that obesity itself may affect the systemic inflammatory state by altering adipokines and gastrointestinal hormone secretion, and that weight loss has been shown to lead to remission of diabetes mellitus (33), this could potentially impact the results of this study. (6) The NHANES data used in this study came from the U.S. population, which makes the generalization of the findings regionally restrictive.

## 5 Conclusion

In summary, this study demonstrates that diets rich in antioxidants exert beneficial effects on DN and renal function in DN, with chronic inflammation potentially playing a mediating role. However, these findings and the underlying mechanisms require further validation and exploration in future research.

## Data Availability

The original contributions presented in the study are included in the article/Supplementary material, further inquiries can be directed to the corresponding authors.

## Glossary

ALB: Albumin
ALT: Alanine aminotransferase
AST: Aspartate aminotransferase
ALP: Alkaline phosphatase
AGEs: Advanced glycation end products
BMI: Body mass index
CDAI: Composite dietary antioxidant index
CKD: Chronic kidney disease
CKD-EPI: Chronic Kidney Disease Epidemiology Collaboration
CRP: C-reactive protein
DOBS: Dietary oxidative balance score
DN: Diabetic nephropathy
DBP: Diastolic blood pressure
DE: Direct effect
eGFR: estimated glomerular filtration rate
ESRD: End-stage renal disease
FBG: Fasting blood glucose
GFR: Glomerular filtration rate
G/L: Granulocyte-to-lymphocyte ratio
HDL-C: High-density lipoprotein cholesterol
IDF: International Diabetes Federation
INFLA-score: Low-grade inflammation score
IE: Indirect effect
LDL-C: Low-density lipoprotein cholesterol
L: Lymphocyte
M: Monocyte
MI: Multiple imputation
NHANES: National Health and Nutritional Examination Survey
NCHS: National Center for Health Statistics
N: Neutrophil
NF-κB: Nuclear factor-κB
OS: Oxidative stress
OR: Odds ratio
PSM: Propensity score matching
RCS: Restricted cubic spline
Ref: Reference
STROBE: Strengthening the Reporting of Observational Studies in Epidemiology
Scr: Serum creatinine
SBP: Systolic blood pressure
TC: Total cholesterol
TG: Triglyceride
TGF-*β*: transforming growth factor-β
TAC: Total antioxidant capacity
UACR: Urine albumin-to-creatinine ratio
VIF: Variance inflation factors
WBC: White blood cell
WTDRD1: Dietary day one sample weight
WTDR2D: Dietary two-day sample weight.
