# Supplementary material for: Effects of dietary oxidative balance score on diabetic nephropathy and renal function: insights from retrospective and cross-sectional studies
Source: Front Nutr. 2025 Mar 20;12:1560913. doi: 10.3389/fnut.2025.1560913 (PMC11965115; doi:10.3389/fnut.2025.1560913)
Supplement: Supplementary file 1 [file Table_1.DOCX]

**eFigure 1.** The balance of the dataset before and after PSM.


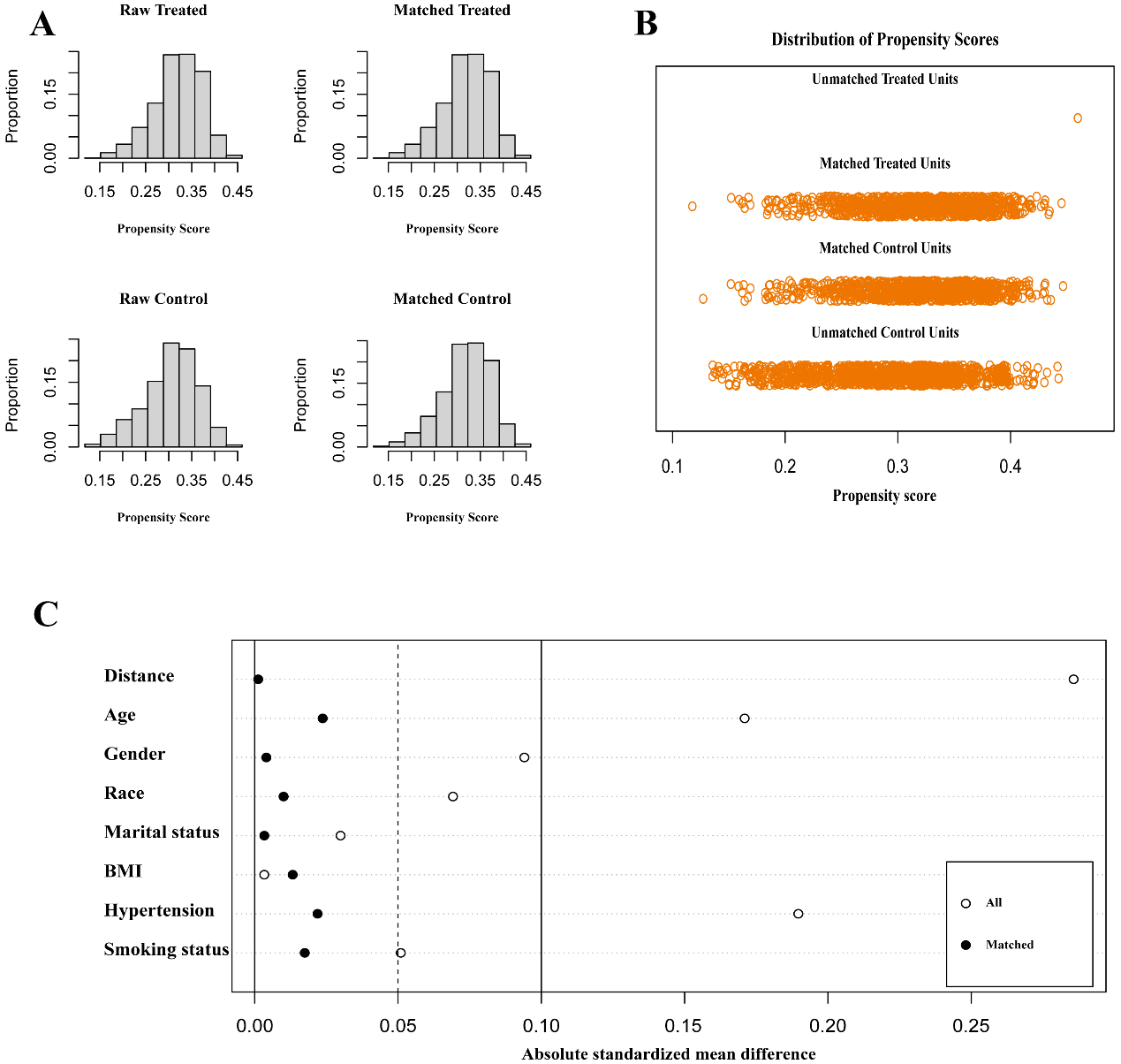


**Notes: A:** The histogram of propensity score distribution of the raw data and the matched data; **B:** The jitter graph of propensity score distribution of raw data and matched data; **C:** The Love plot of absolute standardized mean difference (SMD) of all covariates in raw data and matched data. **Abbreviations:** BMI, Body mass index.

**eTable 1.** Specific calculation steps.

| **DOBS** | (1) Intake of the 16 dietary constituents was divided into sex-specific tertiles;  (2) Based on previous studies,^1^ the score allocation scheme were:  i. Dietary antioxidants (Q1: 0 point; Q2: 1 points; Q3: 2 points);  ii. Dietary pro-oxidants (Q1: 2 points; Q2: 1 points; Q3: 0 point). |
| --- | --- |
| **INFLA-score^2^** | (1) 10-tiles of each parameter levels;  (2) the highest deciles (7-10) were scored 1 to 4 points, respectively;  (3) the lowest dectiles (1-4) were scored -4 to -1 points, respectively;  (4) the dectiles 5 or 6 was scored 0 points. |

**Abbreviations:** DOBS, Dietary oxidative balance score; Ualb, Urine albumin; Ucr, Urine creatinine; UACR, Urine albumin-to-creatinine ratio; Scr, Serum creatinine; eGFR, estimated glomerular filtration rate; INFLA-score, Low-grade inflammation score;

**Reference:**

1. Zhang W, Peng SF, Chen L, Chen HM, Cheng XE, Tang YH. Association between the Oxidative Balance Score and Telomere Length from the National Health and Nutrition Examination Survey 1999-2002. Oxid Med Cell Longev. 2022;2022:1345071. doi:10.1155/2022/1345071

2. Bonaccio M, Di Castelnuovo A, Pounis G, et al. A score of low-grade inflammation and risk of mortality: prospective findings from the Moli-sani study. Haematologica. 2016;101(11):1434-1441. doi:10.3324/haematol.2016.144055

**eTable 2.** DOBS assignment scheme.

| **DOBS components** | **Property** | **Male** | | | **Female** | | |
| --- | --- | --- | --- | --- | --- | --- | --- |
|  |  | **0** | **1** | **2** | **0** | **1** | **2** |
| **Before PSM (n = 3218)** | |  |  |  |  |  |  |
| Dietary fiber, g/day | A | < 12.200 | 12.200-18.750 | ≥ 18.750 | < 10.750 | 12.200-18.750 | ≥ 15.850 |
| Carotene, ug/day | A | < 624.00 | 624.00-2288.0 | ≥ 2288.0 | < 691.50 | 624.00-2288.0 | ≥ 2251.5 |
| Vitamin B2, mg/day | A | < 1.6475 | 1.6475-2.3785 | ≥ 2.3785 | < 1.3235 | 1.6475-2.3785 | ≥ 1.8910 |
| Niacin, mg/day | A | < 18.475 | 18.475-27.017 | ≥ 27.017 | < 14.757 | 18.475-27.017 | ≥ 20.863 |
| Vitamin B6, mg/day | A | < 1.4430 | 1.4430-2.1560 | ≥ 2.1560 | < 1.2135 | 1.4430-2.1560 | ≥ 1.7430 |
| Total folate, ug/day | A | < 291.00 | 291.00-447.00 | ≥ 447.00 | < 245.50 | 291.00-447.00 | ≥ 360.00 |
| Vitamin B12, ug/day | A | < 3.4000 | 3.4000-5.9100 | ≥ 5.9100 | < 2.5750 | 3.4000-5.9100 | ≥ 4.4450 |
| Vitamin C, mg/day | A | < 38.950 | 38.950-92.450 | ≥ 92.450 | < 40.900 | 38.950-92.450 | ≥ 90.550 |
| Vitamin E, mg/day | A | < 4.7400 | 4.7400-7.6700 | ≥ 7.6700 | < 3.9300 | 4.7400-7.6700 | ≥ 6.1900 |
| Calcium, mg/day | A | < 605.00 | 605.00-944.00 | ≥ 944.00 | < 528.00 | 605.00-944.00 | ≥ 814.50 |
| Magnesium, mg/day | A | < 224.50 | 224.50-316.00 | ≥ 316.00 | < 189.00 | 224.50-316.00 | ≥ 260.50 |
| Zinc, mg/day | A | < 8.6450 | 8.6450-13.550 | ≥ 13.550 | < 6.8200 | 8.6450-13.550 | ≥ 10.010 |
| Copper, mg/day | A | < 0.9880 | 0.9880-1.4060 | ≥ 1.4060 | < 0.8325 | 0.9880-1.4060 | ≥ 1.1360 |
| Selenium, ug/day | A | < 86.150 | 86.125-126.25 | ≥ 126.25 | < 68.000 | 86.125-126.25 | ≥ 96.450 |
| Total fat, g/day | P | ≥ 89.345 | 59.380-89.345 | < 59.380 | ≥ 87.600 | 59.380-89.345 | < 58.305 |
| Iron, mg/day | P | ≥ 17.505 | 11.675-17.505 | < 11.675 | ≥ 17.280 | 11.675-17.505 | < 11.450 |
| **After PSM (n = 1994)** | |  |  |  |  |  |  |
| Dietary fiber, g/day | A | < 12.000 | 12.200-18.300 | ≥ 18.300 | < 10.500 | 10.500-15.500 | ≥ 15.500 |
| Carotene, ug/day | A | < 614.00 | 614.00-2192.0 | ≥ 2192.0 | < 655.00 | 655.00-2195.5 | ≥ 2195.5 |
| Vitamin B2, mg/day | A | < 1.6150 | 1.6150-2.3625 | ≥ 2.3625 | < 1.3085 | 1.3085-1.8810 | ≥ 1.8810 |
| Niacin, mg/day | A | < 18.095 | 18.095-26.277 | ≥ 26.277 | < 14.408 | 14.408-20.577 | ≥ 20.577 |
| Vitamin B6, mg/day | A | < 1.3840 | 1.3840-2.1170 | ≥ 2.1170 | < 1.2120 | 1.2120-1.7470 | ≥ 1.7470 |
| Total folate, ug/day | A | < 284.00 | 284.00-437.50 | ≥ 437.50 | < 239.50 | 239.50-358.00 | ≥ 358.00 |
| Vitamin B12, ug/day | A | < 3.2800 | 3.2800-5.8700 | ≥ 5.8700 | < 2.5600 | 2.5600-4.4900 | ≥ 4.4900 |
| Vitamin C, mg/day | A | < 38.600 | 38.600-91.550 | ≥ 91.550 | < 40.100 | 40.100-90.300 | ≥ 90.300 |
| Vitamin E, mg/day | A | < 4.5850 | 4.5850-7.3900 | ≥ 7.3900 | < 3.6950 | 3.6950-6.1400 | ≥ 6.1400 |
| Calcium, mg/day | A | < 588.50 | 588.50-942.50 | ≥ 942.50 | < 516.00 | 516.00-799.00 | ≥ 799.00 |
| Magnesium, mg/day | A | < 218.00 | 218.00-308.00 | ≥ 308.00 | < 185.50 | 185.50-254.00 | ≥ 254.00 |
| Zinc, mg/day | A | < 8.4550 | 8.4550-13.340 | ≥ 13.340 | < 6.6700 | 6.6700-9.8700 | ≥ 9.8700 |
| Copper, mg/day | A | < 0.9675 | 0.9675-1.3720 | ≥ 1.3720 | < 0.8120 | 0.8120-1.1220 | ≥ 1.1220 |
| Selenium, ug/day | A | < 84.100 | 84.100-124.15 | ≥ 124.15 | < 66.700 | 66.700-95.450 | ≥ 95.450 |
| Total fat, g/day | P | ≥ 89.345 | 59.380-89.345 | < 59.380 | ≥ 65.375 | 42.235-65.375 | < 42.235 |
| Iron, mg/day | P | ≥ 17.505 | 11.665-17.505 | < 11.665 | ≥ 13.890 | 9.2570-13.890 | < 9.2750 |
| **Afetr MI (n = 3380)** |  |  |  |  |  |  |  |
| Dietary fiber, g/day | A | < 12.200 | 12.200-18.650 | ≥ 18.650 | < 10.800 | 10.800-15.585 | ≥ 15.850 |
| Carotene, ug/day | A | < 607.00 | 607.00-2225.0 | ≥ 2225.0 | < 676.00 | 676.00-2224.0 | ≥ 2224.0 |
| Vitamin B2, mg/day | A | < 1.6485 | 1.6485-2.3845 | ≥ 2.3840 | < 1.3220 | 1.3220-1.8910 | ≥ 1.8910 |
| Niacin, mg/day | A | < 18.386 | 18.386-26.997 | ≥ 26.997 | < 14.623 | 14.623-20.898 | ≥ 20.898 |
| Vitamin B6, mg/day | A | < 1.4370 | 1.4370-2.1540 | ≥ 2.1540 | < 1.2120 | 1.2120-1.7415 | ≥ 1.7415 |
| Total folate, ug/day | A | < 292.00 | 292.00-448.00 | ≥ 448.00 | < 246.00 | 246.00-361.00 | ≥ 361.00 |
| Vitamin B12, ug/day | A | < 3.4000 | 3.4000-5.9300 | ≥ 5.9300 | < 2.5600 | 2.5600-4.4200 | ≥ 4.4200 |
| Vitamin C, mg/day | A | < 39.400 | 39.400-93.200 | ≥ 93.200 | < 40.900 | 40.900-90.800 | ≥ 90.800 |
| Vitamin E, mg/day | A | < 4.7550 | 4.7550-7.6250 | ≥ 7.6250 | < 3.9100 | 3.9100-6.1800 | ≥ 6.1800 |
| Calcium, mg/day | A | < 608.50 | 608.50-948.00 | ≥ 948.00 | < 526.00 | 526.00-813.50 | ≥ 813.50 |
| Magnesium, mg/day | A | < 223.50 | 223.50-316.00 | ≥ 316.00 | < 189.00 | 189.00-260.00 | ≥ 260.00 |
| Zinc, mg/day | A | < 8.6450 | 8.6450-13.605 | ≥ 13.605 | < 6.7950 | 6.7950-10.060 | ≥ 10.060 |
| Copper, mg/day | A | < 0.9825 | 0.9825-1.4020 | ≥ 1.4020 | < 0.8310 | 0.8310-1.1385 | ≥ 1.1385 |
| Selenium, ug/day | A | < 85.850 | 85.850-125.75 | ≥ 125.75 | < 67.700 | 67.700-96.700 | ≥ 96.700 |
| Total fat, g/day | P | ≥ 89.130 | 59.500-89.130 | < 59.500 | ≥ 66.250 | 43.830-66.250 | < 43.830 |
| Iron, mg/day | P | ≥ 17.505 | 11.660-17.505 | < 11.660 | ≥ 14.020 | 9.4000-14.020 | < 9.4000 |

**Notes:** A means antioxidant; B means pro-oxidant. **Abbreviations:** DOBS, Dietary oxidative balance score; PSM, Propensity score matching; MI, Multiple imputation.

**eTable 3.** Baseline characteristics of participants based on hospital physical examination center.

| Clinical variables | Total  (n = 124) | DN  (n = 62) | Non-DN  (n = 62) | *P* |
| --- | --- | --- | --- | --- |
| Age | 65.00 (55.75, 74.00) | 64.50 (56.00, 72.25) | (55.25, 74.00) | 0.843 |
| Gender |  |  |  | 0.362 |
| Male | 73 (58.87%) | 39 (62.90%) | 34 (54.84%) |  |
| Female | 51 (41.13%) | 23 (37.10%) | 28 (45.16%) |  |
| BMI, kg/m^2^ | 29.20 (24.98, 33.38) | 29.30 (24.93, 33.20) | 28.90 (25.10, 33.72) | 0.790 |
| ALB, g/L | 42.00 (40.00, 44.00) | 42.00 (40.00, 44.00) | 42.00 (40.00, 45.00) | 0.977 |
| ALT, U/L | 23.00 (17.00, 34.00) | 23.50 (18.25, 35.50) | 22.50 (16.00, 33.00) | 0.400 |
| AST, U/L | 24.00 (18.75, 30.25) | 23.00 (18.00, 28.75) | 24.50 (19.00, 31.00) | 0.494 |
| ALP, U/L | 71.00 (58.50, 89.25) | 69.00 (55.00, 93.00) | 72.50 (62.00, 88.00) | 0.507 |
| TC, mmol/L | 4.75 ± 1.05 | 4.69 ± 0.95 | 4.82 ± 1.14 | 0.492 |
| TG, mmol/L | 1.56 (1.18, 2.36) | 1.58 (1.23, 2.17) | 1.56 (1.14, 2.48) | 0.937 |
| HDL-C, mmol/L | 1.14 (0.98, 1.32) | 1.12 (0.96, 1.32) | 1.16 (1.01, 1.36) | 0.514 |
| LDL-C, mmol/L | 2.74 ± 0.94 | 2.70 ± 0.88 | 2.79 ± 1.00 | 0.605 |
| Ca, mmol/L | 2.35 (2.27, 2.40) | 2.35 (2.30, 2.40) | 2.35 (2.27, 2.42) | 0.855 |
| Fe, umol/L | 14.30 (11.30, 17.95) | 14.80 (11.30, 17.50) | 13.35 (11.30, 18.32) | 0.716 |
| Zn, umol/L | 13.39 ± 2.04 | 13.66 ± 2.27 | 13.11 ± 1.76 | 0.135 |
| Cu, umol/L | 17.98 (15.77, 20.95) | 17.49 (15.93, 20.42) | 18.67 (15.21, 21.74) | 0.909 |
| Se, umol/L | 1.69 (1.60, 1.88) | 1.75 (1.63, 1.91) | 1.63 (1.55, 1.82) | 0.002 |
| eGFR, ml/min | 83.66 ± 23.39 | 90.64 ± 16.68 | 76.68 ± 26.95 | < 0.001 |
| Hypertension |  |  |  | 0.556 |
| Yes | 87 (70.16%) | 42 (67.74%) | 45 (72.58%) |  |
| No | 37 (29.84%) | 20 (32.26%) | 17 (27.42%) |  |
| Smoke |  |  |  | 0.857 |
| Yes | 65 (52.42%) | 33 (53.23%) | 32 (51.61%) |  |
| No | 59 (47.58%) | 29 (46.77%) | 30 (48.39%) |  |

**Abbreviations:** DN, Diabetics nephropathy; BMI, Body mass index; ALB, Albumin; ALT, Alanine aminotransferase; AST, Aspartate aminotransferase; ALP, Alkaline phosphatase; TC, Total cholesterol; TG, Triglyceride; HDL-C, High-density lipoprotein cholesterol; LDL-C, Low-density lipoprotein cholesterol; eGFR, estimated glomerular filtration rate.

**eTable 4.** Single-factor and multifactor logistic regression analysis of serum microelements and DN.

| Clinical variables | Single-factor logistic regression | | Multifactor logistic regression | |
| --- | --- | --- | --- | --- |
|  | OR (95% CI) | *P* | OR (95% CI) | *P* |
| *Ca, mmol/L | 1.035 (0.720, 1.486) | 0.854 | 1.073 (0.723, 1.594) | 0.726 |
| Fe, umol/L | 1.014 (0.941, 1.093) | 0.714 | 1.057 (0.958, 1.165) | 0.271 |
| Zn, umol/L | 0.873 (0.729, 1.044) | 0.137 | 0.864 (0.709, 1.053) | 0.147 |
| Cu, umol/L | 1.005 (0.925, 1.091) | 0.908 | 1.010 (0.912, 1.118) | 0.851 |
| *Se, umol/L | 0.766 (0.641, 0.914) | 0.003 | 0.764 (0.638, 0.917) | 0.004 |

**Notes:** * means the effect size caused by an increase of each 10 units in the independent variable. **Abbreviations:** DN, Diabetics nephropathy; eGFR, estimated glomerular filtration rate; OR, Odds ratio.

**eTable 5.** Correlation analysis between serum microelements and renal function in DN.

| Clinical variables | eGFR (DN) | |
| --- | --- | --- |
|  | *r* | *P* |
| Ca, mmol/L | 0.056 | 0.666 |
| Fe, umol/L | 0.407 | 0.001 |
| Zn, umol/L | 0.303 | 0.017 |
| Cu, umol/L | -0.226 | 0.078 |
| Se, umol/L | 0.296 | 0.020 |

**Abbreviations:** DN, Diabetics nephropathy; eGFR, estimated glomerular filtration rate.

**eTable 6.** Weighted baseline characteristics of participants after PSM.

| **Characteristics** | **All** | **DOBS** | | | | ***P*** |
| --- | --- | --- | --- | --- | --- | --- |
|  |  | **Q1** | **Q2** | **Q3** | **Q4** |  |
| **Continuous variables (Mean ± SD)** | |  |  |  |  |  |
| DOBS | 16.84 ± 6.57 | 7.36 ± 2.00 | 13.17 ± 1.43 | 18.41 ± 1.72 | 24.56 ± 1.99 | < 0.001 |
| Age, years | 60.40 ± 13.72 | 61.47 ± 13.66 | 62.01 ± 13.28 | 59.30 ± 14.03 | 59.48 ± 13.64 | 0.002 |
| BMI, kg/m^2^ | 32.79 ± 7.43 | 32.57 ± 6.84 | 31.94 ± 7.31 | 33.87 ± 7.82 | 32.57 ± 7.44 | < 0.001 |
| Ualb, ug/ml | 212.86 ± 1049.12 | 272.31 ± 1091.91 | 229.47 ± 1517.71 | 223.06 ± 774.35 | 150.81 ± 774.41 | 0.313 |
| Ucr, mg/dl | 115.86 ± 72.81 | 119.54 ± 81.98 | 114.97 ± 74.06 | 120.18 ± 74.22 | 110.06 ± 62.84 | 0.078 |
| UACR, mg/g | 195.04 ± 828.40 | 280.55 ± 957.41 | 204.13 ± 1073.05 | 177.91 ± 678.69 | 145.76 ± 619.46 | 0.081 |
| Scr, mg/dl | 1.00 ± 0.47 | 1.06 ± 0.59 | 1.04 ± 0.56 | 0.99 ± 0.42 | 0.94 ± 0.29 | < 0.001 |
| eGFR, mL/min/1.73m^2^ | 80.82 ± 24.76 | 78.61 ± 25.74 | 78.80 ± 26.22 | 80.94 ± 25.02 | 83.72 ± 22.30 | 0.002 |
| Dietary fiber, g/day | 15.60 ± 8.04 | 8.90 ± 3.49 | 12.58 ± 4.57 | 15.25 ± 5.50 | 22.70 ± 8.74 | < 0.001 |
| Carotene, ug/day | 2433.83 ± 3396.50 | 1212.07 ± 2425.97 | 1924.53 ± 2487.20 | 2383.03 ± 2975.66 | 3687.95 ± 4359.12 | < 0.001 |
| Vitamin B2, mg/day | 2.07 ± 0.96 | 1.22 ± 0.43 | 1.73 ± 0.59 | 2.12 ± 0.71 | 2.85 ± 1.02 | < 0.001 |
| Niacin, mg/day | 22.92 ± 10.34 | 14.06 ± 5.06 | 18.72 ± 6.49 | 23.51 ± 7.67 | 31.51 ± 10.64 | < 0.001 |
| Vitamin B6, mg/day | 1.85 ± 0.99 | 1.03 ± 0.34 | 1.46 ± 0.51 | 1.86 ± 0.63 | 2.69 ± 1.16 | < 0.001 |
| Total folate intake, ug/day | 375.59 ± 177.53 | 211.71 ± 74.13 | 312.38 ± 98.70 | 376.94 ± 124.79 | 532.58 ± 184.40 | < 0.001 |
| Vitamin B12, ug/day | 5.46 ± 7.13 | 2.67 ± 1.81 | 4.10 ± 4.37 | 5.79 ± 9.21 | 8.05 ± 7.88 | < 0.001 |
| Vitamin C, mg/day | 78.75 ± 73.05 | 44.09 ± 49.05 | 65.16 ± 56.92 | 78.77 ± 66.67 | 112.37 ± 87.25 | < 0.001 |
| Vitamin E, mg/day | 6.77 ± 4.52 | 3.50 ± 1.58 | 5.27 ± 2.34 | 6.62 ± 3.48 | 10.23 ± 5.52 | < 0.001 |
| Calcium, mg/day | 825.92 ± 443.32 | 469.42 ± 210.21 | 658.77 ± 298.83 | 861.14 ± 342.16 | 1159.89 ± 481.87 | < 0.001 |
| Magnesium, mg/day | 268.96 ± 111.85 | 156.13 ± 42.90 | 215.30 ± 49.72 | 271.82 ± 65.81 | 382.79 ± 104.40 | < 0.001 |
| Zinc, mg/day | 11.82 ± 9.44 | 6.63 ± 2.74 | 9.27 ± 4.49 | 11.49 ± 4.99 | 17.52 ± 14.09 | < 0.001 |
| Copper, mg/day | 1.30 ± 1.21 | 0.75 ± 0.27 | 1.04 ± 0.67 | 1.38 ± 1.56 | 1.79 ± 1.32 | < 0.001 |
| Selenium, ug/day | 104.22 ± 47.52 | 67.06 ± 23.39 | 85.88 ± 29.39 | 108.26 ± 38.03 | 139.36 ± 52.50 | < 0.001 |
| Total fat intake, g/day | 74.91 ± 36.58 | 50.84 ± 22.72 | 63.65 ± 25.88 | 77.02 ± 32.30 | 97.67 ± 40.61 | < 0.001 |
| Iron, , mg/day | 14.96 ± 7.27 | 8.99 ± 3.36 | 12.29 ± 4.32 | 14.63 ± 4.60 | 21.29 ± 8.10 | < 0.001 |
| Total energy intake, kcal/day | 1869.41 ± 719.58 | 1304.08 ± 436.92 | 1593.54 ± 498.97 | 1905.08 ± 554.60 | 2425.43 ± 739.47 | < 0.001 |
| WBC, 1000 cells/ul | 7.77 ± 2.60 | 7.86 ± 2.46 | 7.77 ± 3.45 | 7.97 ± 2.35 | 7.53 ± 2.09 | 0.034 |
| Lymphocyte,  1000 cells/ul | 2.23 ± 1.44 | 2.27 ± 0.88 | 2.26 ± 2.55 | 2.26 ± 1.02 | 2.17 ± 0.81 | 0.572 |
| Monocyte,  1000 cells/ul | 0.59 ± 0.21 | 0.58 ± 0.19 | 0.59 ± 0.24 | 0.60 ± 0.22 | 0.58 ± 0.20 | 0.328 |
| Neutrophils,  1000 cells/ul | 4.67 ± 1.81 | 4.73 ± 1.88 | 4.67 ± 1.99 | 4.81 ± 1.76 | 4.51 ± 1.64 | < 0.001 |
| Eosinophils,  1000 cells/ul | 0.22 ± 0.17 | 0.23 ± 0.18 | 0.21 ± 0.15 | 0.23 ± 0.17 | 0.23 ± 0.16 | 0.086 |
| Basophils,  1000 cells/ul | 0.05 ± 0.06 | 0.04 ± 0.05 | 0.04 ± 0.06 | 0.05 ± 0.06 | 0.04 ± 0.06 | 0.233 |
| G/L | 2.54 ± 1.41 | 2.43 ± 1.19 | 2.64 ± 1.70 | 2.59 ± 1.32 | 2.48 ± 1.38 | 0.115 |
| Platelet | 259.16 ± 81.62 | 263.56 ± 79.68 | 259.59 ± 91.21 | 264.51 ± 83.95 | 250.95 ± 72.06 | 0.022 |
| CRP, mg/dl | 0.70 ± 1.24 | 0.85 ± 1.87 | 0.67 ± 1.20 | 0.70 ± 0.93 | 0.62 ± 0.92 | 0.030 |
| INFLA-score | 0.65 ± 6.43 | 1.05 ± 6.32 | 0.13 ± 6.44 | 1.52 ± 6.29 | -0.05 ± 6.49 | < 0.001 |
| **Categorical variables (%)** | |  |  |  |  |  |
| Gender |  |  |  |  |  | 0.013 |
| Male | 54.78 | 56.09 | 54.52 | 49.44 | 58.95 |  |
| Female | 45.22 | 43.91 | 45.48 | 50.56 | 41.05 |  |
| Race |  |  |  |  |  | < 0.001 |
| Mexican American | 10.10 | 11.02 | 8.81 | 9.96 | 10.57 |  |
| Non-Hispanic Black | 16.91 | 26.93 | 18.21 | 15.30 | 10.59 |  |
| Non Hispanic White | 61.99 | 49.44 | 59.71 | 66.77 | 67.86 |  |
| Others | 11.00 | 12.60 | 13.27 | 7.98 | 10.98 |  |
| Marital status |  |  |  |  |  | 0.030 |
| Never married | 8.48 | 9.95 | 5.82 | 8.48 | 9.44 |  |
| Married/living with partner | 61.56 | 59.83 | 61.15 | 59.20 | 65.21 |  |
| Windowed/divorced/separated | 29.96 | 30.23 | 33.03 | 32.31 | 25.35 |  |
| Hypertension |  |  |  |  |  | 0.001 |
| No | 12.23 | 10.67 | 10.74 | 8.81 | 17.51 |  |
| Yes | 87.77 | 89.33 | 89.26 | 91.19 | 82.49 |  |
| Smoking status |  |  |  |  |  | < 0.001 |
| Non-smokers | 43.50 | 46.58 | 37.72 | 41.51 | 47.48 |  |
| Former smoker | 36.71 | 29.04 | 40.08 | 40.00 | 36.43 |  |
| Current smoker | 19.79 | 24.37 | 22.19 | 18.49 | 16.09 |  |
| Diabetic nephropathy |  |  |  |  |  | 0.003 |
| No | 53.14 | 45.06 | 53.78 | 56.51 | 55.09 |  |
| Yes | 46.86 | 54.94 | 46.22 | 43.49 | 44.91 |  |

**Abbreviations:** DOBS, Dietary oxidative balance score; Ualb, Urine albumin; Ucr, Urine creatinine; UACR, Urine albumin-to-creatinine ratio; Scr, Serum creatinine; eGFR, estimated glomerular filtration rate; INFLA-score, Low-grade inflammation score; PSM, Propensity score matching.

**eTable 7.** The respective and pooled effect values after MI based on fully adjusted model.

| **Exposure** | **OR/β (95% CI), *P* (Model 3)** | |
| --- | --- | --- |
|  | **Outcome: DN** | **Outcome: eGFR in DN** |
| **MI 1** |  |  |
| DOBS | 0.98 (0.97, 0.99), 0.002 | 0.184 (0.089, 0.278), < 0.001 |
| DOBS (Categorical) |  |  |
| Q1 | Ref | Ref |
| Q2 | 0.90 (0.73, 1.10), 0.304 | 2.249 (0.338, 4.160), 0.021 |
| Q3 | 0.73 (0.59, 0.91), 0.004 | 2.239 (0.354, 4.125), 0.020 |
| Q4 | 0.76 (0.61, 0.94), 0.013 | 3.687 (1.803, 5.571), < 0.001 |
| *P* for trend | 0.003 | 0.004 |
| **MI 2** |  |  |
| DOBS | 0.98 (0.97, 0.99), 0.002 | 0.185 (0.091, 0.280), < 0.001 |
| DOBS (Categorical) |  |  |
| Q1 | Ref | Ref |
| Q2 | 0.89 (0.72, 1.10), 0.296 | 2.253 (0.342, 4.164), 0.021 |
| Q3 | 0.73 (0.59, 0.90), 0.004 | 2.245 (0.359, 4.131), 0.020 |
| Q4 | 0.76 (0.61, 0.94), 0.013 | 3.720 (1.836, 5.605), < 0.001 |
| *P* for trend | 0.003 | 0.004 |
| **MI 3** |  |  |
| DOBS | 0.98 (0.97, 0.99), 0.002 | 0.183 (0.088, 0.277), < 0.001 |
| DOBS (Categorical) |  |  |
| Q1 | Ref | Ref |
| Q2 | 0.90 (0.73, 1.10), 0.304 | 2.235 (0.323, 4.147), 0.022 |
| Q3 | 0.73 (0.59, 0.90), 0.004 | 2.203 (0.317, 4.089), 0.022 |
| Q4 | 0.76 (0.61, 0.94), 0.013 | 3.665 (1.779, 5.551), < 0.001 |
| *P* for trend | 0.003 | 0.005 |
| **MI 4** |  |  |
| DOBS | 0.98 (0.97, 0.99), 0.002 | 0.183 (0.088, 0.278), < 0.001 |
| DOBS (Categorical) |  |  |
| Q1 | Ref | Ref |
| Q2 | 0.89 (0.73, 1.10), 0.298 | 2.243 (0.331, 4.155), 0.022 |
| Q3 | 0.73 (0.59, 0.91), 0.004 | 2.202 (0.316, 4.088), 0.022 |
| Q4 | 0.76 (0.61, 0.95), 0.014 | 3.674 (1.789, 5.559), < 0.001 |
| *P* for trend | 0.004 | 0.005 |
| **MI 5** |  |  |
| DOBS | 0.98 (0.97, 0.99), 0.001 | 0.184 (0.089, 0.279), < 0.001 |
| DOBS (Categorical) |  |  |
| Q1 | Ref | Ref |
| Q2 | 0.89 (0.72, 1.10), 0.297 | 2.248 (0.336, 4.160), 0.021 |
| Q3 | 0.73 (0.59, 0.90), 0.004 | 2.215 (0.329, 4.102), 0.021 |
| Q4 | 0.76 (0.61, 0.94), 0.012 | 3.686 (1.801, 5.572), < 0.001 |
| *P* for trend | 0.003 | 0.005 |
| **Combined effect values** |  |  |
| DOBS | 0.98 (0.97, 0.99), < 0.001 | 0.184 (0.089, 0.279), < 0.001 |
| DOBS (Categorical) |  |  |
| Q1 | Ref | Ref |
| Q2 | 0.89 (0.73, 1.10), 0.291 | 2.246 (0.334, 4.157), 0.021 |
| Q3 | 0.73 (0.59, 0.90), 0.004 | 2.221 (0.334, 4.107), 0.021 |
| Q4 | 0.76 (0.61, 0.94), 0.013 | 3.686 (1.801, 5.572), < 0.001 |

**Abbreviations:** DOBS, Dietary oxidative balance score; DN, Diabetics nephropathy; eGFR, estimated glomerular filtration rate; Ref, Reference; OR, Odds ratio.

**eTable 8.** Subgroup analysis of DOBS and DN and eGFR in DN before PSM.

| **Characteristics** | **OR or β (95% CI), *P*** | ***P* for interaction** |
| --- | --- | --- |
| **Outcome 1*** |  |  |
| Gender |  | 0.278 |
| Male | 0.98 (0.96, 0.99), 0.005 |  |
| Female | 0.99 (0.98, 1.01), 0.345 |  |
| Race |  | 0.301 |
| Mexican American | 0.99 (0.97, 1.01), 0.310 |  |
| Non-Hispanic Black | 0.98 (0.96, 1.00), 0.136 |  |
| Non Hispanic White | 0.98 (0.96, 1.00), 0.019 |  |
| Others | 1.01 (0.97, 1.04), 0.692 |  |
| Marital status |  | 0.138 |
| Never married | 1.03 (0.99, 1.07), 0.216 |  |
| Married/living with partner | 0.98 (0.97, 1.00), 0.009 |  |
| Windowed/divorced/separated | 0.98 (0.96, 1.00), 0.086 |  |
| Hypertension |  | 0.517 |
| No | 0.98 (0.95, 1.01), 0.204 |  |
| Yes | 0.98 (0.97, 1.00), 0.013 |  |
| Smoking status |  | 0.461 |
| Non-smokers | 0.98 (0.96, 1.00), 0.028 |  |
| Former smoker | 0.98 (0.96, 1.00), 0.077 |  |
| Current smoker | 1.00 (0.97, 1.02), 0.894 |  |
| **Outcome 2#** |  |  |
| Gender |  | 0.112 |
| Male | 0.383 (0.144, 0.621), 0.002 |  |
| Female | 0.041 (-0.324, 0.407), 0.825 |  |
| Race |  | 0.084 |
| Mexican American | 0.870 (0.421, 1.318), < 0.001 |  |
| Non-Hispanic Black | 0.456 (-0.121, 1.032), 0.123 |  |
| Non Hispanic White | 0.037 (-0.255, 0.330), 0.802 |  |
| Others | 0.681 (0.099, 1.263), 0.024 |  |
| Marital status |  | 0.708 |
| Never married | 0.462 (-0.203, 1.126), 0.177 |  |
| Married/living with partner | 0.160 (-0.104, 0.424), 0.236 |  |
| Windowed/divorced/separated | 0.412 (0.016, 0.809), 0.043 |  |
| Hypertension |  | 0.390 |
| No | -0.117 (-0.578, 0.344), 0.620 |  |
| Yes | 0.278 (0.052, 0.503), 0.016 |  |
| Smoking status |  | 0.552 |
| Non-smokers | 0.164 (-0.166, 0.494), 0.330 |  |
| Former smoker | 0.287 (-0.061, 0.635), 0.107 |  |
| Current smoker | 0.449 (0.029, 0.870), 0.038 |  |

**Notes:** *: Outcome is DN; #: Outcome is eGFR in DN. **Abbreviations:** DOBS, Dietary oxidative balance score; DN, Diabetics nephropathy; eGFR, estimated glomerular filtration rate; PSM, Propensity score matching; OR, Odds ratio.

**eTable 9.** Subgroup analysis of DOBS and DN and eGFR in DN after PSM.

| **Characteristics** | **OR or β (95% CI), *P*** | ***P* for interaction** |
| --- | --- | --- |
| **Outcome 1*** |  |  |
| Gender |  | 0.461 |
| Male | 0.98 (0.96, 1.00), 0.039 |  |
| Female | 0.99 (0.97, 1.01), 0.565 |  |
| Race |  | 0.095 |
| Mexican American | 1.01 (0.98, 1.03), 0.721 |  |
| Non-Hispanic Black | 0.98 (0.95, 1.01), 0.117 |  |
| Non Hispanic White | 0.97 (0.95, 1.00), 0.021 |  |
| Others | 1.01 (0.97, 1.06), 0.551 |  |
| Marital status |  | 0.528 |
| Never married | 1.03 (0.98, 1.08), 0.293 |  |
| Married/living with partner | 0.98 (0.97, 1.00), 0.082 |  |
| Windowed/divorced/separated | 0.98 (0.96, 1.01), 0.145 |  |
| Hypertension |  | 0.416 |
| No | 0.98 (0.94, 1.02), 0.248 |  |
| Yes | 0.99 (0.97, 1.00), 0.078 |  |
| Smoking status |  | 0.271 |
| Non-smokers | 0.99 (0.97, 1.01), 0.314 |  |
| Former smoker | 0.97 (0.95, 1.00), 0.030 |  |
| Current smoker | 1.00 (0.97, 1.03), 0.999 |  |
| **Outcome 2#** |  |  |
| Gender |  | 0.102 |
| Male | 0.377 (0.138, 0.615), 0.002 |  |
| Female | 0.026 (-0.336, 0.389), 0.888 |  |
| Race |  | 0.076 |
| Mexican American | 0.862 (0.408, 1.317), < 0.001 |  |
| Non-Hispanic Black | 0.436 (-0.134, 1.006), 0.135 |  |
| Non Hispanic White | 0.017 (-0.275, 0.309), 0.909 |  |
| Others | 0.690 (0.110, 1.270), 0.021 |  |
| Marital status |  | 0.664 |
| Never married | 0.506 (-0.165, 1.177), 0.144 |  |
| Married/living with partner | 0.145 (-0.118, 0.408), 0.282 |  |
| Windowed/divorced/separated | 0.382 (-0.014, 0.778), 0.060 |  |
| Hypertension |  | 0.326 |
| No | -0.125 (-0.574, 0.324), 0.587 |  |
| Yes | 0.267 (0.042, 0.493), 0.021 |  |
| Smoking status |  | 0.552 |
| Non-smokers | 0.152 (-0.175, 0.480), 0.363 |  |
| Former smoker | 0.263 (-0.085, 0.610), 0.139 |  |
| Current smoker | 0.438 (0.017, 0.859), 0.043 |  |

**Notes:** *: Outcome is DN; #: Outcome is eGFR in DN. **Abbreviations:** DOBS, Dietary oxidative balance score; DN, Diabetics nephropathy; eGFR, estimated glomerular filtration rate; PSM, Propensity score matching; OR, Odds ratio.

**eTable 10.** Threshold effect analysis of DOBS and DN and eGFR in DN.

| **Outcome** | **DN**  **OR (95% CI), *P*** | | **eGFR in DN**  **β (95% CI), *P*** | |
| --- | --- | --- | --- | --- |
|  | **Before PSM** | **After PSM** | **Before PSM** | **After PSM** |
| **Model 1** |  |  |  |  |
| Linear effect | 0.98 (0.97, 1.00), 0.006 | 0.99 (0.97, 1.00), 0.038 | 0.254 (0.047, 0.461), 0.016 | 0.238 (0.031, 0.444), 0.024 |
| **Model 2** |  |  |  |  |
| Inflection point (K) | 26 | 26 | 25 | 18 |
| DOBS < K | 0.99 (0.98, 1.00), 0.048 | 0.99 (0.98, 1.00), 0.160 | 0.172 (-0.058, 0.401), 0.143 | -0.131 (-0.693, 0.431), 0.648 |
| DOBS > K | 0.80 (0.63, 1.03), 0.082 | 0.82 (0.64, 1.06), 0.134 | 2.436 (-0.234, 5.106), 0.074 | 0.446 (0.086, 0.806), 0.015 |
| Log likelihood ratio | 0.095 | 0.160 | 0.106 | 0.164 |

**Abbreviations:** DOBS, Dietary oxidative balance score; DN, Diabetics nephropathy; eGFR, estimated glomerular filtration rate; PSM, Propensity score matching; OR, Odds ratio.
